# Investigating the implementation of differentiated HIV services and implications for pregnant and postpartum women: A mixed methods multi-country study

**DOI:** 10.1080/17441692.2020.1795221

**Published:** 2020-07-29

**Authors:** Rujeko S. Chimukuche, Alison Wringe, John Songo, Farida Hassan, Lameck Luwanda, Thoko Kalua, Mosa Moshabela, Jenny Renju, Janet Seeley

**Affiliations:** a Africa Health Research Institute, KwaZulu-Natal, South Africa; b London School of Hygiene and Tropical Medicine, London, UK; c Malawi Epidemiology & Intervention Research Unit, Karonga, Malawi; d Ifakara Health Institute, Dar es Salaam, Tanzania; e Department of HIV and AIDS, Ministry of Health, Lilongwe, Malawi; f University of KwaZulu-Natal, Durban, South Africa

**Keywords:** HIV, differentiated service delivery, ART delivery, sub-Saharan Africa, HIV policy implementation

## Abstract

Universal antiretroviral therapy (ART) for pregnant and postpartum women in sub-Saharan Africa has required adaptations to service delivery. We compared national policies on differentiated HIV service delivery with facility-level implementation, and explored provider and user experiences in rural Malawi, Tanzania and South Africa. Four national policies and two World Health Organization guidelines on HIV treatment for pregnant and postpartum women published between 2013 and 2017 were reviewed and summarised. Results were compared with implementation data from surveys undertaken in 34 health facilities. Eighty-seven in-depth interviews were conducted with pregnant and post-partum women living with HIV, their partners and providers. In 2018, differentiated service policies varied across countries. None specifically accounted for pregnant or postpartum women. Malawian policies endorsed facility-based multi-month scripting for clinically-stable adult ART patients, excluding pregnant or breastfeeding women. In Tanzania and South Africa, national policies proposed community-based and facility-based approaches, for which pregnant women were not eligible. Interview data suggested some implementation of differentiated services for pregnant and postpartum women beyond stipulated policies in all settings. Although these adaptations were appreciated by pregnant and postpartum women, they could lead to frustrations among other users when criteria for fast-track services or multi-month prescriptions were not clear.

## Background

In 2013, the World Health Organisation (WHO) recommended lifelong antiretroviral therapy (ART) for all pregnant women living with HIV, regardless of their health status (known as Option B+) (World Health Organisation, 2013). By 2015, the recommendation was expanded to cover all adults living with HIV (World Health Organisation, 2015). This approach, widely known as Universal Test and Treat (UTT), was rapidly adopted by most Eastern and Southern African countries, including South Africa, Tanzania and Malawi in 2016 (UNAIDS, 2018). In the Eastern and Southern African region, UTT implementation has led to growing numbers of people living with HIV (PLHIV) receiving care and treatment, with an estimated 12.9 million people accessing antiretroviral therapy in 2017, representing 66% of those living with HIV (UNAIDS, 2018). Among pregnant and postpartum women living with HIV in Eastern and Southern Africa, ART coverage increased from 47 to 93% between 2010 and 2017 (UNAIDS, 2018).

Whilst this increase in the proportion of PLHIV who are on ART bodes well for reaching international targets for ending the AIDS epidemic by 2030 (World Health Organisation, 2017a), growing evidence suggests that it has put health systems under increasing pressure (Duncombe et al., 2015). In some settings, the expansion of ART programmes has exacerbated shortages of staff (Nachega et al., 2016), leading to overcrowded HIV clinics and long waiting times for patients attending for drug refills (Idindili et al., 2018). In other contexts, supply chains have not been able to keep up with increasing demands, leading to stock-outs of HIV test kits and antiretroviral drugs (Gils et al., 2018). These strains on the health system may undermine service quality, as overstretched healthcare workers struggle to keep up to date with newly revised protocols and procedures associated with evolving HIV treatment policies (Duncombe et al., 2015; Kwesigabo et al., 2012).

There is growing agreement that a ‘one-size-fits-all’ model of HIV service delivery will not succeed in providing sustainable access to ART for all PLHIV (Grimsrud et al., 2016). This has led to a call for differentiated service delivery (DSD), a term that has been coined to describe HIV services that are patient-centred, simplified and adapted to include the various preferences and expectations of PLHIV, whilst reducing the burden on the health system (Grimsrud et al., 2016). In 2016, WHO proposed a differentiated care framework that included four elements that specified the types of services delivered (What), the location of the service delivery (Where), the providers of the service (Who) and the frequency of the service delivery (When) (World Health Organisation, 2017b). The content of these components may vary across settings, but each DSD model aims to improve ART accessibility and support retention in care, to promote sustained viral suppression among people living with HIV (UNAIDS, 2014; World Health Organisation, 2016). The adoption of differentiated ART delivery models, and associated implementation research, has accelerated over the past five years (Ehrenkranz et al., 2019; Grimsrud et al., 2017). Several models have emerged locally in response to context-specific health systems challenges. For example, in Mozambique, community adherence groups developed as a means to enable PLHIV to obtain ART refills in communities that were located far away from the clinics (Decroo et al., 2011). Health care worker groups have been created for patients attending clinics in rural and informal settlements in Eswatini and Kenya as a way to address high patient volumes and long waiting times (Khabala et al., 2015; Pasipamire et al., 2018). Mobile outreach ART clinics and home delivery services have also been implemented in Eswatini in an effort to increase retention in care among PLHIV who experience challenges in reaching ART clinics (Pasipamire et al., 2018). In Malawi, appointment spacing for clinically stable patients has been adopted to enable health workers to spend more time with patients with more complex clinical needs (Cawley et al., 2016).

Initially pregnancy was considered an exclusion criterion for differentiated models of ART delivery for clinically stable clients (International AIDS Society, 2016). However, in 2017, WHO published consolidated ART guidelines that included the recommendation that in some cases, children, pregnant and post-partum women and other members of key populations could be managed as clinically-stable clients. However, it is not clear to what extent these recommendations have been adopted in national policies, and how HIV services are being delivered in practice for pregnant and post-partum women in different settings. Understanding how to optimise ART delivery for pregnant and postpartum women is particularly important in view of the large body of evidence demonstrating poor retention rates among women who initiate ART during pregnancy (Srivastava et al., 2018).

The aim of this paper is to describe national policies on DSD in Malawi, Tanzania and South Africa, and to explore the formal and informal policy adaptation in the implementation of HIV services delivered to women within prevention of mother-to-child transmission (PMTCT) programmes in three rural African settings.

## Methods

### Study design

Data for this analysis were drawn from the study for Strengthening Health Systems for the Application of Policy to Enable Universal Test and Treat (SHAPE UTT), undertaken in health and demographic surveillance sites (HDSS) in South Africa, Malawi and Tanzania, with the aim of assessing the health systems impacts of Universal Test and Treat (authors forthcoming). This analysis draws on reviews of national HIV policies, health facility surveys and qualitative data collected through in-depth interviews with health service users, their partners and healthcare providers in each setting between 2018 and 2019.

### Study settings

In Malawi, the study was conducted in Karonga district, a rural area in the north of the country where most residents are subsistence farmers. HIV prevalence in Karonga district is 10.5% for women and 8.7% for men (National Statistics Office, 2017). ART has been available to the population since 2004 through decentralisation to small health facilities and district hospitals. In 2011, the Malawian Ministry of Health adopted Option B+, followed by UTT in 2016 (National Statistics Office, 2017).

In Tanzania, the study was implemented in Ifakara, where adult HIV prevalence is 7% (Church et al., 2017). ART scale-up in Tanzania started in 2004 at the district hospitals, and was later decentralised to primary health facilities (Letang et al., 2017; Levira et al., 2015). The government adopted Option B+ in 2013 and UTT in 2016.

In South Africa, the study was conducted in uMkhanyakude district where the adult HIV prevalence rate was 33% in 2017 (South Africa Department of Health, 2015a), substantially higher than the national average of 14% (South Africa Department of Health, 2017b). The country has experienced rapid policy changes relating to the delivery and provision of HIV services over the past decade. In 2015, South Africa adopted Option B+ for pregnant women, with UTT following shortly afterwards in 2016 (South Africa Department of Health, 2015b).

### Policy reviews

WHO recommendations and national policy documents on differentiated service care delivery were retrieved through online searches of websites of national HIV programmes. Data on policy content and publication date were summarised and organised in Excel.

### Facility survey

In each country, a survey was conducted in all the health facilities serving the populations of each HDSS in 2017-2018 (Table 1). The facility survey has been described in detail elsewhere (Church et al., 2017). In brief, the questionnaire included modules on the delivery of HIV testing and counselling services, antenatal services, and ART services. The survey was administered in English to the person in charge at the health facility. Data were entered into MS SQL Server (Microsoft Corp, Redmond, USA), and subsequently cleaned and exported for descriptive analysis using Excel.

### Qualitative methods

#### Sampling and recruitment of participants

The study population for differentiated service delivery for pregnant and post-partum women included women using antenatal care or HIV services, their partners and HIV service providers. Other populations such as partners and health service providers were included in the sample because they provided broader insights into the differences in implementation of DSD policies across the three sites. Female service users were pregnant women living with HIV who initiated ART during their current pregnancy, antenatal care users (HIV-negative or unknown HIV status), postnatal women living with HIV attending routine ART clinics, or women lost to follow up (LTFU) during their current pregnancy having initiated ART. These participants were sampled through PMTCT service registers, with trained field workers approaching participants at the clinics and recruiting them for interviews. LTFU registers and routine tracing systems were used to approach participants who were no longer engaged in care. Partners of women who were pregnant or postpartum and had initiated ART through Option B+ were sampled in uMkhanyakude and Ifakara and were recruited through the female service users who participated in the study, following their consent. Health providers were sampled from the clinics serving the study populations or associated with district-level management of PMTCT delivery, ensuring a range of cadres and roles in delivering ART services to pregnant and postpartum women.

### Data collection

Semi-structured topic guides for in-depth interviews with service users included questions on their experiences of HIV services that they had received. Topic guides for health providers explored their experiences of delivering HIV services, including their perspectives on issues that influenced implementation.

Topic guides were translated into the local language and back translated. Following informed consent, in-depth interviews were conducted in local languages from October 2017 to December 2018. In uMkhanyakude, interviews were conducted in isiZulu, the first language of the participants. In Karonga, interviews were conducted in Chitumbuka and in Ifakara, Kiswahili was used. In all sites, interviews were conducted by trained by social science research assistants, in private settings and lasted approximately forty-five minutes to an hour. In-depth interviews were audio-recorded where consent was provided, and otherwise detailed notes were taken. Interview summaries were written by the fieldworkers after each interview. Debriefings between the lead social scientist researchers and the research assistants in each site were conducted after each interview to consider emerging findings. Audio-recordings were transcribed and translated into English. Data quality checks were conducted by the facilitators to ensure completeness and accuracy of transcripts.

### Data analysis and interpretation

Data analysis was done by the lead social scientists at each site. In-depth interview transcripts were coded manually using Excel spreadsheets in uMkhanyakude and aided by Nvivo version 11 in Karonga and Ifakara. In all sites, the transcripts were initially reviewed with a focus on the broad areas pertaining to experiences of receiving or delivering HIV services. Codes relating to the experiences of health service users and providers were then inductively generated from their accounts. Initial themes and hypotheses emerging through the analysis process were then generated and explored further in the data. Comparisons were made across the different settings. Direct quotations of health service users and providers are used to illustrate the findings.

### Ethical approval

Ethical approval for the data collection was obtained from the University of KwaZulu-Natal Biomedical Research Committee in South Africa (BE400/14), from the National Institute of Medical Research, in Tanzania (NIMR/HQ/R.8a/Vol. IX /2579) and from the National Health Sciences Research Committee in Malawi (17/07/1861). The study was also approved by the ethics committee at the London School of Hygiene and Tropical Medicine (13536).

## Results

We present the differentiated service delivery policies adopted by each country during the study period using the Where, Who, When framework (International AIDS Society, 2016). We then assess implementation of these policies within the study areas using the facility survey data. Finally, we explore the experiences of the providers and users who delivered and received these services in each setting.

### Policy review

The 2017 WHO guidance on differentiated service delivery models for key populations, families and pregnant women was not adopted in any of the three study countries (World Health Organisation, 2017b). However, South Africa and Tanzania adopted DSD policies in 2016 and 2017 respectively, with differentiated service delivery models for the general adult population in alignment to those recommended by WHO (Ministry of Health Community Development Gender Elderly and Children, 2017; South Africa Department of Health, 2015a, 2016, 2017a). In Malawi, multi-month scripting (of up to 3 months) was included in the 2016 ART guidelines (Ministry of Health Malawi, 2016) following evidence that appointment-spacing reduced patient volumes in clinics (Wringe et al., 2018), but policy documents describing differentiated service delivery approaches more generally had not been produced by 2018 (Table 2).

In South Africa, spaced and fast-lane appointment systems for stable patients, adherence clubs, and a central chronic medication dispensing and distribution (CCMDD) system have been endorsed in national policy documents from 2014 (Meyer et al., 2017; South Africa Department of Health, 2016; Wringe et al., 2018). In Tanzania, the 2017 national policy on the management and treatment of HIV allowed facility-based individual fast-track services, health worker-managed groups and community-level ART groups (Ministry of Health Community Development Gender Elderly and Children, 2017).

### Implementation of differentiated care service delivery policies

We assessed facility-level implementation of the differentiated service delivery policies adopted by each country (Table 3). The policy review was led by the first author, a social scientist. Policy documents were retrieved online for each of the countries. Differentiated HIV service delivery policies and guidelines were extracted and collated in an excel spreadsheet.

In Ifakara, 75% of facilities reported that other people could collect ART for patients only under exceptional circumstances, representing a deviation from policy which explicitly stated that ‘treatment supporters’ could be designated to collect ART refills on a patient’s behalf. The policy on who can collect drug refills in South Africa was not explicit and was absent from Malawian policy. In 18% of facilities in uMkhanyakude and 60% of facilities in Karonga, a third party could collect drugs on behalf of patients, but not at every visit, and only one pre-designated person could undertake this collection (76% of facilities in uMkhanyakude and 20% of facilities in Karonga).

In Ifakara and uMkhanyakude 58% and 59% respectively of health facilities stated that ART refills were usually provided for one month, representing a deviation from policy in both settings. In Karonga 60% of facilities reported providing multi-month prescriptions for three months, in line with policy, while 40% of facilities provided refills for two months.

Policies in South Africa and Tanzania explicitly allowed for the use of facility-based pharmacies to provide ART refills to alleviate clinic overcrowding, while this was not mentioned in Malawian policies. However, most facilities in all sites reported that ART refills were only provided at ART clinics. Policies in South Africa mentioned the role of community-based distribution outlets with 12% of facilities in uMkhanyakude reporting additional community-based ART refill options, and 18% reporting that refills were provided to health worker managed patient groups. In Ifakara, all facilities reported that ART refills were provided in clinics with no community-based options reported, in line with national policy.

### Experiences of delivering and receiving differentiated service delivery models

In total, 87 participants were interviewed regarding their experiences of delivering or receiving HIV services (Table 4).

### Who

In Ifakara and uMkhanyakude, health care workers and clients described how HIV services were only delivered by healthcare providers, with no patient-managed models. In Karonga, volunteers reportedly delivered ART supplies to patients’ homes when they were too sick to attend the clinics and in Ifakara, health workers assist by giving medication to patients thereby facilitating their adherence and reducing the burden of drug collection for their caregivers:

The male volunteer carried medicine and delivered to me at home. When I reached home, I was taken from my house to be nursed at my co-wife’s house because I had no one to cook for me at my house. When the volunteer came home with drugs at home he gave it to my husband. My husband kept drugs on my behalf and gave me to take while I was going to sleep in the evening (Female service user, aged 34, Karonga, Malawi).

There is a time I came, I was still pregnant, there was no medicine. I have close relation with one nurse here, my mother called her and she gave me the medicine which was for the staff. (Female service user, aged 25, Ifakara, Tanzania)

### When

Multi-month prescriptions were perceived to be an exception to the rule by patients and health workers in all settings.

In uMkhanyakude, one pregnant young woman who was a student reported that she was able to receive extended doses of ART medication to enable her to go and look for a school. This flexibility meant that the student had enough pills and felt encouraged to stay on ART treatment:

In January, I was supposed to go and look for a school. Then I told them that I won’t be here. I will come back in March for delivery. Then they gave me double [supply of drugs] so that when I come back in March I will still have them. Because I was unable to start school in January.. I had to come back home. I still had my pills as well (Female service user, aged 19, uMkhanyakude, South Africa).

In Ifakara, there were some reports that patients wanting prescriptions for longer periods of time had to make special requests:

I told the nurse I will be travelling. So, I requested for extra, I will be travelling away and will come late. For February and March (Female service user, aged 38, Ifakara, Tanzania).

In some cases, it appeared that long-standing relationships and trust built between patients and health providers enabled the provider to be confident to trust patients with additional ART supplies.

In all settings, some patients were unclear as to the criteria or processes for receiving multiple months of their drug supply. One client thought the health worker was being sympathetic to them by giving them more months of ART refill containers, while he was unable to give any explanation for the apparent differences in prescription durations:

I may not know what the agreement was before, as you may find others receiving three containers, while others receive one container. Because when they come to me, they just ask me if I receive one container, then I would just say yes, I received one. So, I think there is an agreement that was made before, saying that this person receives three containers and I am only allowed to receive one container as they normally ask me if I received one container. And maybe the others when they started, they received three containers so that is why they still receive three (Male partner, aged 33, uMkhanyakude, South Africa).

In uMkhanyakude, some women reported that HIV postpartum women could be given more than one container after their child’s immunisation visits at the health facility:

They said once I am done with the baby’s immunization, then I will go back to receiving two containers as I was receiving two containers before as I was at xxx (Female service user, aged 28, uMkhanyakude, South Africa)

Fast lane approaches were introduced in some facilities in uMkhanyakude and Ifakara sites, to reduce waiting times. In some instances, these could lead to anger and concern among some service users due to a lack of understanding as to why there was apparently preferential treatment for HIV positive pregnant women to go in front of other patients when there were lengthy queues:

Everybody is on one queue. Sick people and pregnant people. But sometimes they just call the pregnant people to come forward. Yes, the ones that are sick. (Female service user, aged 23, uMkhanyakude, South Africa).

Sometimes, they have to start with pregnant women. Sometimes, others followed by us. That is a challenge. For example, they should have installed a special system for us. Like, when we arrive, they arrange a time for us like how that nurse was doing. When we were arriving in the morning, she attended us before other people. After finishing with us, she shifted to others. In that regard, it was not easy for other people to know what is going on. (Female service user, aged 38, Ifakara, Tanzania).

### Where

Location of service delivery was not indicated in Tanzania and Malawi, however, in uMkhanyakude in South Africa, some participants mentioned their experiences of alternative locations for service provision. Many health workers appreciated the significant reduction in their workload since the creation of community ART refill clubs that were facilitated by community caregivers (CCGs), who are lay health workers employed by the department of health:

Yes, there are some clubs that formed. We’ve been assisted with CCGs - yah they do the treatment here with the nurse, … … ….Yah it is a relief because they are so many, those ones, they are about 30 to 50. Yes, you see that it is helping, and they are getting two months treatment (Male health worker, uMkhanyakude, South Africa).

Health care workers and users attributed the success of ART refill clubs to the fact that CCGs are recognised by community members as trusted health providers. Furthermore, they are trusted by the formal health care staff, and have emerged as an instrumental force underlying the implementation of ART adherence clubs in this setting. From health care worker perspectives, good monitoring systems provided by these CCGs ensure that the ART refill groups are run efficiently.

However, despite this success, some health care workers in uMkhanyakude were frustrated by their perception that patients were not properly utilising alternative options for care delivery, including fast track lanes, therefore limiting their utility in alleviating congestion in clinics:

CCMDD is the way of decanting number of patients, those patients who are viral supressed who are well who doesn’t have like TB or communicable disease we enrol them on CCMDD.…. It’s only that some of the patients we enrol them on CCMDD, but they still pull the files and follow the queue. They are not supposed to pull the file, they are supposed to take their appointment cards, go inside the clinic and give the appointment card. They don’t have to follow the queue to the pharmacist, the pharmacist will give a parcel and the patient goes (Female health worker, uMkhanyakude, South Africa).

Furthermore, despite options for ART refill collection available in uMkhanyakude, providers explained that many patients still preferred to collect their refills in the clinics:

It just that most patients here in rural areas they don’t like to go to town because they prefer their parcels to be delivered here in the clinic. Why they don’t like going to town? Because of transport fee (Female health worker, uMkhanyakude, South Africa).

In this case, additional travel costs associated with going to the external pick up points negated any potential benefits.

## Discussion

Our findings suggest that there were similarities and differences in the adoption of policies in the three rural settings in South Africa, Malawi and Tanzania. Across all three countries, the 2017 WHO recommendations for differentiated antiretroviral therapy delivery for specific populations, including pregnant and post-partum women, had not been systematically adopted into national policies by 2018. Differences in DSD policy endorsements could be noticed in the three countries, where early adoption was seen in South Africa and Tanzania in 2016 and 2017 respectively but not explicitly stated in Malawi. Malawi included multi-month scripting of three months in the 2016 ART guidelines but had not produced policy documents on DSD by 2018.

However, various adaptations to ART service delivery for pregnant and post-partum women were being made in practice in each setting. In some instances, a lack of clarity among HIV service users over the eligibility criteria or rationale for some patients to apparently be able to access differentiated models of care, seen as preferential, caused confusion and frustration.

The facility level adaptations to ART service delivery for pregnant women that were observed in our study, despite their mention in national policies, could help some women to overcome barriers to accessing HIV services, and reduce the workload of health workers, as found elsewhere for the general adult population (Rasschaert et al., 2014; Selke et al., 2010; Smith et al., 2014). Our findings suggest that such adaptations can lead to more flexible services for pregnant and post-partum women.

However, we also found that some patients in uMkhanyakude did not use fast-track queues, even when they were in place, which may suggest that the procedures were not clear, or that patients preferred the system that they were used to, or wanted more face-to-face time with clinicians and nurses. Further research is merited to explore these issues in this setting. A reluctance to use fast-track queues by patients may be attributed to some clients still preferring to receive regular HIV care in clinical settings where they have established nurse/client relationships, receive regular health screening and can connect to other clients despite waiting and spending more time at the clinic (Sharer et al., 2019). Research from elsewhere in South Africa has also shown that post-partum women may avoid differentiated models of care if they are not in close proximity to health services for their infants (Myer et al., 2017).

We also observed sub-optimal implementation of current DSD policies in each country. For example, providers in Ifakara reported not allowing other specified people to collect ART supplies for their client, and facilities in Ifakara and uMkhanyakude also reported only providing a one-month supply of drugs, despite the national policy for multi-month scripting. Other studies have highlighted that flexible HIV care policies can lead to varied implementation (Ambia et al., 2017; Jones et al., 2019).

Additionally, our findings suggest that some patients may need to have sufficient confidence in their rapport with providers to request greater flexibility in the duration of their ART prescriptions. Health workers may need more detailed guidance on how to implement these patient-centred policies and patients themselves need more information on the different models of care that are available, as well as the criteria for eligibility, and processes for accessing them, to ensure that patient needs are accommodated. Furthermore, additional care is needed to ensure that ‘eligibility’ does not become a value-based reward system. For example, the South African policy indicates that facilities may implement decongestion strategies to ‘reward adherent and stable chronic patients’ with flexibility in the collection of their medication. Greater flexibility being provided to stable patients as a reward risks disadvantaging those patients who may benefit most from differentiated care options to help them to address the barriers that underlie their non-adherence to ART. Those barriers may include long travelling distances, high transportation costs, long waiting times in health facilities, issues of stigma and poor attitudes of some healthcare workers towards clients (Adjetey et al., 2019).

The data on which this paper is based is drawn from the Strengthening Health Systems for the Application of Policy to Enable Universal Test and Treat (SHAPE UTT) study. That study focused on broad aspects of the impact of Option B+ on health systems, not exclusively on adaptations to HIV service delivery for pregnant and post-partum women. While a strength of this study was our ability to draw on data from three countries, a limitation was that the interview topic guides were not tailored to focus on differentiated care service delivery. Therefore, we drew our findings from data provided on the broader impact of Option B+ policies on HIV service delivery.

## Conclusion

We found that despite an absence of national policies on differentiated service delivery for pregnant and post-partum women in Tanzania, Malawi and South Africa during the study period, there were various facility-level adaptations to service delivery that were implemented in the different settings. Comparisons across the sites showed that there were localised variations in the way different settings adapted and implemented service delivery. These adaptations could facilitate HIV care engagement, but non-consistency in their application, and a lack of clarity over procedures and eligibility could undermine trust in HIV services by different service users.

## Figures and Tables

**Table 1:** Type of facilities surveyed by site.

	South Africa	Malawi	Tanzania
*HDSS site name*	uMkhanyakude	Karonga	Ifakara
*Size of HDSS site (km^2^)*	438	135	2400
*Population of HDSS site*	90,000	42,555	169,000
*HIV prevalence in the HDSS*	35.2%	9.6%	7%
*No. of facilities included in the survey*	17	5	12
*Type of facility*			
Small clinic/dispensary	16	0	3
Large clinic/small health centre	1	2	0
Large health centre/sub-district hospital	0	3	6
District/referral hospital	0	0	0

**Table 2:** Summary of differentiated HIV service delivery policies in each country.

	WHO Guidelines	South Africa	Malawi	Tanzania
Who	Trained and supervised lay providers can distribute ART to adults, adolescents and children living with HIV. Trained and supervised community health workers can dispense ART between regular clinic visits^1^ For women diagnosed and initiating ART during pregnancy, clinical consultations and ART refills should be integrated into maternal, newborn and child health care where clinical consultations managed by health or lay workers^e^ ART refills collected by individuals either at facilities or at other distribution points or by groups of clients (in a community ART distribution model)^1^	Health facilities need to implement decongestion strategies that ‘reward adherent and stable^a^ chronic patients’ with a faster service and flexibility to choose their preferred medication collection service’^b,c^	Only the patient or his registered guardians/ treatment supporter is allowed to collect ARVs^6^	Facility-based health worker managed groups: clients are seen in age-specific groups managed by a health care worker, e.g. teen clubs, youth clubs^d^ Family member or treatment supporter refills – clients proposes a family member or treatment supporter to collect their ART refill from the pharmacy^5^
When	‘Multi-month prescribing’ whereby depending on the HIV treatment and patient eligibility guidelines in a country, ART patients may receive several months of ART drugs at one time, and will not need to return to health facilities and/or clinics monthly to receive their ARV supply^1^ People-centred care offers a patient appointment system and frequency of facility visits	Spaced and fast lane appointments are established as a repeat prescription collection strategy.^4^ A minimum of 3 months drug supply and optimised prescription periods to meet the needs of key and vulnerable populations and improve adherence^4^	Stable patients receive refills of ART for three or more months instead of one month at a time, so stable patients have a maximum of four clinic visits per year instead of 12. Stable and adherent patients can be given up to 12-week (3-month) appointments^f^	ART refills for stable clients should be provided for two months depending on supply^5^
Where	Initiation of ART can happen at peripheral health facilities with maintenance at the community level (Community level includes external outreach sites, health posts, home-based services or community-based organisations)^g^ Through existing outreach services, community-based organisations can provide support for out-of-facility individual models of ART delivery: for example, through regular outreach to hotspots^1^ ART refills should be provided as close to people’s homes as possible. Consideration could be given to delinking refill collection from maternal, newborn and child health care^1^ Consideration can be given to using lay providers equivalent to mentor mothers for task shifting ART refill distribution^1^	Roll-out of innovative approaches to increase treatment uptake and improve treatment outcomes that include male- and adolescent-friendly clinic hours; community- or home-based ART initiation, community facilitated ART refill clubs^3^ A centralised dispensing operation that obtains prescriptions for stable chronic patients from health facilities and dispenses a package of medicine for each patient, which they can collect from a health facility (using an express queue) or another convenient pick up point e.g at pharmacies^4^	ARVs may not be further distributed outside of certified ART health facilities^6^	Facility based Individual fast-track from pharmacy - clients are seen individually within health care facilities and are fast-tracked for collection of the ARVs^5^ ART refill should be decentralised to existing facilities including primary health facilities at existing PMTCT sites. Additional dispensaries and health centres should be facilitated to be ART sites dependant on local demand and a clinic access assessment^5^ ART refills can be delivered through a mobile outreach strategy by health care worker in hard to reach areas. Clients are seen individually outside of health care facilities by clinical staff as part of routine outreach^5^

a **Stable patient:** The definition of a stable patient put forward by the World Health Organisation in the 2016 guidelines, states that these are people who have received ART for at least one year and have no adverse drug reactions that require regular monitoring, no current illnesses or pregnancy, are not currently breastfeeding, have good understanding of lifelong adherence and evidence of treatment success (that is two consecutive viral load measurements below 1000 copies/mL and in the absence of viral load monitoring, rising CD4 cell counts or CD4 counts >200 cells/mm^3^ can be used to indicate treatment success).

b South Africa Department of Health (2016).

c South Africa Department of Health (2017a).

d Ministry of Health Community Development Gender Elderly and Children (2017).

e World Health Organisation (2017b).

f Ministry of Health Malawi (2016).

g World Health Organisation (2016).

**Table 3:** Differentiated care models implemented by site.

		uMkhanyakude		Ifakara		Karonga	
		Total	%	Total	%	Total	%
**Who**	**Can ART be collected by other people**						
	* Never*	0	0%	0	0%	0	0%
	* In exceptional circumstances*	1	6%	9	75%	1	20%
	* Yes by anyone*	0	0%	2	17%	0	0%
	* yes but not every visit*	3	18%	0	0%	3	60%
	* Yes by one designated person*	13	76%	1	8%	1	20%
**When**	**Frequency of ART refill visits**						
	* Monthly*	10	59%	7	58%	0	0%
	* Every 2 months*	7	41%	4	33%	2	40%
	* Every 3 months*	0	0%	1	8%	3	60%
**Where**	**Where can patients get ART refills at the clinic**						
	* Clinic only*	15	88%	11	92%	5	100%
	* Pharmacy*	1	6%	1	8%	0	0%
	* HCW managed PLHIV group*	3	18%	0	0%	0	0%
	* Patient managed PLHIV group*	0	0%	0	0%	0	0%
	**Where can patients get ART refills in their community**						
	* Not available*	10	59%	12	100%	5	100%
	* community pharmacy*	2	12%	0	0%	0	0%
	* pat manage adherence group*	3	18%	0	0%	0	0%
	* HCW manage adherence group*	3	18%	0	0%	0	0%

**Table 4:** Group participants interviewed per study site.

Sample Groups	South Africa uMkhanyakude	Malawi Karonga	Tanzania Ifakara	Total
*PMTCT B+ service users*	8	5	8	21
*HIV Routine service users*	6	7	4	17
*PMTCT Lost to follow up*	6	5	2	13
*Partners*	6	0	9	15
*Health care workers*	7	7	7	21
*Refusals*	0	0	0	0
*Total*	33	24	30	87
